# Aspiration and silent aspiration in Niemann-Pick disease type C1: longitudinal findings from the NIH natural history study

**DOI:** 10.1186/s13023-026-04543-8

**Published:** 2026-08-22

**Authors:** Beth I. Solomon, Andrea M. Munoz, Aiden Borruso, Ninet Sinaii, Nicole Farhat, Derek Alexander, Desiree A. Labor, An Dang Do, Forbes D. Porter

**Affiliations:** 1 Speech-Language Pathology Section, Rehabilitation Medicine Department, Mark O. Hatfield Clinical Center, National Institutes of Health, Bethesda, MD USA; 2 Division of Translational Medicine, Eunice Kennedy Shriver National Institute of Child Health and Human Development, National Institutes of Health, Bethesda, MD USA; 3 Biostatistics and Clinical Epidemiology Service, NIH Clinical Center, National Institutes of Health, Bethesda, MD USA

**Keywords:** Niemann–Pick disease type C1, Dysphagia, Aspiration, Silent aspiration, Neurodegenerative, Miglustat, NPC1 Neurologic Severity Score, Seizures, Swallowing

## Abstract

**Background:**

Niemann–Pick disease type C1 (NPC1) is a rare neurodegenerative disorder in which dysphagia is common and aspiration pneumonia is a leading cause of mortality. Aspiration may be clinically apparent or silent, reflecting impaired airway sensation and protective reflexes. We aimed to characterize aspiration and silent aspiration in the National Institutes of Health (NIH) NPC1 Natural History Study (NCT00344331). We analyzed 130 participants with NPC1 enrolled between 2006 and 2024 who underwent speech-language pathology swallowing evaluations, including clinically indicated videofluoroscopic swallow studies (VFSS). Aspiration risk was rated using the NIH Penetration–Aspiration Scale (NIH-PAS). Silent aspiration was defined as aspiration on VFSS without cough, throat clearing, or wet vocal quality. Longitudinal models examined the presence of silent aspiration alone or (2) a NIH-PAS score > 1 (moderate–profound aspiration) or silent aspiration. Covariates included demographics, neurologic disease onset and duration, body weight/BMI, reflux and seizure medications, miglustat use, NPC Neurological Severity Score (NSS) 5-domain total and subscores (including swallow and respiratory), and the Annualized Severity Increment Score (ASIS).

**Results:**

Silent aspiration occurred in 24/130 (18.4%) participants. Among these, 50% demonstrated recurrent silent aspiration at subsequent visits, and 58.3% exhibited intermittent silent aspiration on follow-up. Considering aspiration risk, 27/130 (20.8%) had NIH-PAS > 1, including all silent aspirators. Silent aspiratiors alone had worse NIH-PAS, higher total NSS with poorer swallow, speech, ambulation, and fine-motor subscores, and higher ASIS. Patients with silent aspiration or NIH-PAS > 1 had an increased aspiration risk with longer neurologic symptom duration, seizure history, higher NSS, higher ASIS, and in children with lower weight percentile. Miglustat use was protective in the overall cohort, but not in the pediatric subgroup (< 20 years-old). Mean time to silent aspiration was ~ 10 years from neurologic symptom onset, with no difference by neurological disease onset.

**Conclusion:**

Silent aspiration is common in NPC1 and closely linked to advancing neurologic disease severity than to age-of-onset phenotype. Bedside clinical evaluations may underestimate silent aspiration; proactive longitudinal swallowing surveillance is warranted in individuals with higher NSS/ASIS scores, seizure history, pediatric weight decline, or evolving motor-speech impairment. These findings support incorporating overt and silent aspiration alongside swallowing function, into routine care and therapeutic trials.

## Background

Niemann-Pick Disease type C1 (NPC1) is a rare neurodegenerative disease, typically diagnosed in childhood, with an estimated incidence of 1.12/100,000 live births [1]. Classically, NPC1 clinical manifestations begin in the neonatal period or in infancy with visceral signs such as hepatosplenomegaly, jaundice, and subsequent neurological symptoms that may include developmental delay, vertical gaze palsy, gait imbalance, ataxia, dysarthria, dysphagia, cataplexy, or seizures [2–4]. However, despite overlap in genetic variations and age of neurological onset, each patient progresses differently [5–7]. Diagnosis is often extremely delayed in cases without significant neonatal liver disease or when only a psychiatric component is present as physicians may not be familiar with NPC1 and its wide range of presentation [8]. To date, genetic sequencing is commonly utilized to confirm the diagnosis of NPC1 by identifying disease-causing variants in *NPC1* or *NPC2*. Pathological variants in either of these genes result in the dysfunction of cholesterol trafficking and consequent accumulation of unesterified cholesterol within the late endosome/lysosome of the cell [1].

The U.S. Food and Drug Administration (FDA) recently marked a significant advancement in treating NPC1 by approving arimoclomol (in combination with miglustat) and levacetylleucine, complementing other investigational therapies available [9, 10]. Prior to the FDA approval of recent treatments, miglustat, a glucosylceramide synthase inhibitor, was often prescribed off-label as a standalone therapy since it has been associated with the stabilization of swallowing function, survival, and other skill sets in NPC1 [11–14]. This is noteworthy as dysphagia affects 55–80% of NPC1 patients [3, 15], and is a known risk factor for aspiration pneumonia, accounting for approximately 2/3 of deaths within this population [15].

Our previous research and interpretative analysis of the NPC1 swallowing phenotype revealed the importance of swallowing dysfunction and aspiration risk; however, we did not address forms of aspiration. We have learned that NPC1 swallowing difficulties vary, with aspiration often being a paramount concern for medical professionals, patients, and families. Aspiration is commonly defined as the entry of food, liquid, or oral secretion into the trachea below the true vocal folds, resulting in noticeable symptoms such as coughing and throat clearing in an attempt to expel the material from the respiratory tract [16]. Although not commonly acknowledged, aspiration can occur silently without any overt signs of swallowing difficulty, raising concerns for other potentially associated morbidities. In this paper, we aimed to analyze aspiration and silent aspiration within an NPC1 cohort and describe associated disease characteristics [17, 18].

## Methods

### Population

We evaluated 130 individuals with a confirmed diagnosis of NPC1 who enrolled in the National Institutes of Health (NIH) IRB-approved NPC1 natural history study (NCTT00001367) (06-CH-0086) from 2006 to 2024. Together, these individuals accounted for 468 visits. Informed consent was obtained from either particpants or their guardians. Assent was obtained when applicable based on age and cognitive ability. From this group of 130 patients, we included 450 visits in which a swallowing evaluation had been conducted, enabling us to investigate the presence of aspiration and silent aspiration. Participants who did not undergo swallowing examinations or had telehealth visits had these visits excluded from the analysis. Participants with NPC1 were categorized according to the neurological age-of-onset groups previously reported in our swallowing publications into four groups: no neurological onset, early childhood onset (ECO, 0–6 years; corresponding to the conventional infantile classification), late childhood onset (LCO, 6–15 years; corresponding to the conventional juvenile classification), and adolescent/adult onset (AO, ≥ 15 years; corresponding to the conventional adult classification).

### Niemann-Pick disease assessments

Under the NIH NPC1 protocol, all participants underwent extensive historical and physical examinations, as well as swallowing assessments. Historical information from parental reports and physical examination data were utilized by the primary team to determine the NPC Neurological Severity Score (NSS), which serves as an indicator of a participant’s severity and covers disease-specific aspects. The NSS score is derived from these comprehensive evaluations and serves as an essential indicator of the patient’s severity [19]. In our analysis, we utilized the abbreviated NSS 5-domain scale (ambulation, fine motor, cognition, speech, and swallow), along with the minor respiratory domain from the NSS 17-domain scale. The NPC NSS quantifies concurrent neurological disease burden. Additionally, we incorporated the Annualized Severity Increment Score (ASIS), which considers both the patient’s severity and age at the time of visit to provide an index of disease severity [20].

### Comprehensive swallowing examination

Patients were examined by a speech-language pathologist (SLP) to assess speech and swallowing function. Swallowing evaluations began with a SLP reviewing the patient’s history and the patient completing a 43-item swallowing questionnaire that helps identify any swallowing dysfunction concerns [21]. If swallowing dysfunction concerns were identified from the initial screening; videofluroscopic swallow studies (VFSS) were conducted. The VFSS was conducted to obtain a detailed visual assessment of the oropharyngeal pathophysiology and to identify any aspiration with three different textures (liquids, purees, and solids). A 39-item impairment level analysis performed by the SLP at the time of the study and a post hoc interpretive analysis was applied utilizing the NIH-penetration aspiration scale (NIH-PAS), a five-point Likert scale that categorizes aspiration from 0 (normal) to 4 (profound) [21, 22].

### Modeling

Two longitudinal statistical models were developed to better characterize silent aspiration over time. In the first model, silent aspiration was examined in isolation utilizing a binary definition, defined as aspiration occurring with any texture in the absence of overt clinical signs on VFSS, including cough, throat clearing, or wet vocal quality [23]. The second model expanded this approach by evaluating silent aspiration in conjunction with the NIH-PAS, summarized across three textures, enabling identification of individuals who aspirated without protective responses while incorporating aspiration severity. This combined outcome included participants with NIH-PAS scores greater than 1, with scores of 2, 3, and 4 representing moderate, severe, and profound aspiration, respectively. Specifically, a score of 2 reflected consistent airway entry of contrast on one or more textures, a score of 3 indicated severe aspiration, and a score of 4 denoted profound aspiration.

### Statistics

Data were described using frequency distributions (percentage) and simple descriptive statistics (median, inter-quartile range). We performed a cross-sectional analysis of participant and disease characteristics at baseline, concentrating on whether participants developed silent aspiration at any time point during the study. Continuous data were compared between the two groups using the Wilcoxon rank-sum test, and categorical data were compared using the Fisher’s exact test or tests for trend if the outcome was ordinal. Survival analysis was carried out using the Kaplan-Meier survival estimates, and survival curves between ECO (< 6 years) and LCO (≥ 6 and < 15 years) were compared using the Log rank test. Longitudinal analyses were carried out using generalized linear models for the outcome of silent aspiration, accounting for pertinent covariates. Odds ratios and 95% CI were calculated for disease-specific characteristics in each model. Baseline characteristics considered in the analyses included demographic variables (sex), body weight (kilograms), BMI (kilogram per meters squared), use of reflux and seizure medications, and treatment with miglustat. In addition, several NPC-specific clinical measures were incorporated, including age at neurological onset, neurological onset grouping, five-domain NSS, NSS-Respiratory subscore, ASIS, and NIH-PAS values. These variables were selected to provide a comprehensive clinical profile and to explore potential associations with the onset and progression of NPC1. Analyses were performed using SAS v9.4 (SAS Institute, Inc, Cary, NC).

## Results

### Patient cohort

A total of 130 patients with NPC1 were included in the analysis. Tables 1 and 2 summarize their baseline demographic and clinical characteristics. Patients were categorized according to disease stage as pre-neurological (no manifested neurological symptoms), early clinical onset (ECO), late clinical onset (LCO), or advanced onset (AO). Overall, patients had a mean of 3.4 ± 3.6 study visits during the observation period, with 56 patients (43.1%) completing at least one follow-up visit. The mean follow-up duration was 3.0 ± 4.1 years.

### Modeling based on patients with silent aspiration

We first assessed the presence or absence of silent aspiration with any texture at any time point during study enrollment based on VFSS findings (Table 1). Of the 130 patients, 24 (18.4%) had at least one documented episode of silent aspiration, while 106 (81.6%) did not. Among those with silent aspiration, 12 patients (50%) experienced recurrent episodes on all subsequent follow-up visit, with 7 (58.3%) demonstrating silent aspiration on some follow-up evaluations.

Among the 24 patients with silent aspiration, 13 (54.2%) were ECO, 10 (41.7%) were LCO, and 1 (4.2%) was AO. In comparison, among the 106 patients without silent aspiration, 6 (4.6%) not yet manifested neurological symptoms, 53 (40.8%) were ECO, 32 (24.6%) were LCO, and 15 (11.5%) were AO. No statistically significant differences were found in the distribution of neurological age of onset between those with no silent aspiration at any point in time compared to those with silent aspiration at any point in time (*p* = 0.49).

There were also no significant differences between groups with and without silent aspiration with respect to sex, weight, BMI, use of seizure medications, miglustat use, or NSS respiratory domain (Table 1). Individuals taking reflux or gastrointestinal medications may have an increased risk of silent aspiration (*p* = 0.05).

In contrast, several outcome measures showed statistically significant differences between patients with and without silent aspiration. These included NIH-PAS, NSS 5-domain total score, NSS swallow domain, NSS speech domain, NSS fine motor domain, NSS ambulation domain, and ASIS score.

### Modeling based on patients with a NIH-PAS > 1 and silent aspiration

In a secondary analysis, we identified 27 patients (20.8%) with a NIH-PAS score of 2, 3, or 4—indicating varying degrees of aspiration, which may or may not be silent (Table 2). The remaining 103 patients did not exhibit any form of aspiration (silent or overt) based on VFSS with several textures. Of the 27 patients with NIH-PAS > 1, 24 overlapped with those previously identified as silent aspirators, while 3 additional patients had NIH-PAS > 1 without observed silent aspiration.

Among the 27 patients with NIH-PAS > 1 and silent aspiration, 15 (55.6%) had ECO, 11 (40.7%) had LCO, and 1 (3.7%) had AO. In comparison, among the 103 patients with NIH-PAS scores ≤ 1, 6 (5.8%) were neurologically asymptomatic, 51 (49.5%) had ECO, 31 (30.1%) had LCO, and 15 (14.6%) had AO. No significant differences were identified in neurological onset distribution (*p* = 0.34).

No statistically significant differences were observed between groups with respect to sex, age of neurological onset, weight, BMI, use of reflux/gastrointestinal medications, seizure medications, miglustat use, or NSS respiratory domain (Table 1). However, several clinical measures did show statistically significant differences between groups, including NIH-PAS, NSS 5-domain score, NSS swallow domain, NSS speech domain, NSS fine motor domain, NSS ambulation domain, and ASIS score.

### Longitudinal analysis of NPC1-specific characteristics

**Fig. 1:**
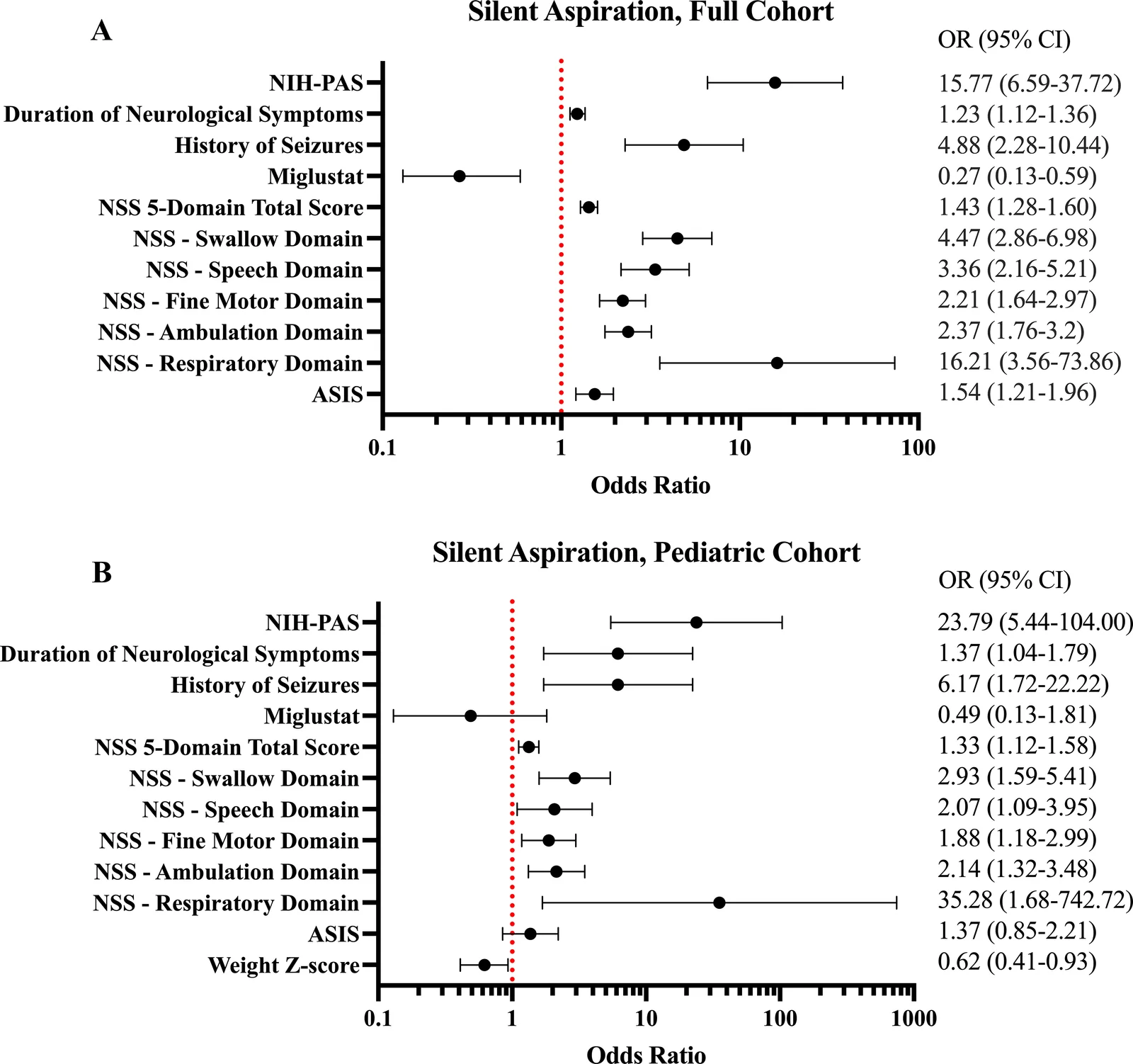
Longitudinal associations between NPC1 characteristics and silent aspiration. **A** represents the full cohort, and **B** represents the pediatric cohort. In the pediatric cohort, weight z-scores reflect age- and sex-standardized body weight relative to normative population references, with lower values indicating poorer nutritional status. Forest plots display adjusted odds ratios (ORs) with 95% confidence intervals derived from longitudinal models assessing the probability of silent aspiration. Legend: Annualized Severity Increment Score (ASIS), NIH-penetration aspiration scale (NIH-PAS), Niemann-Pick Disease type C1 (NPC1), Niemann Pick Severity Scale (NSS), Odds Ratio (OR)

Longitudinal modeling of NPC1-specific characteristics was conducted to assess the probability of silent aspiration (Fig. 1). Longer duration of neurological symptoms, seizure history, and worsening scores in the NSS total (5-domain), swallow, speech, fine motor, ambulation, and respiratory domains, along with higher ASIS scores, were significantly associated with increased odds of silent aspiration (Fig. 1A). Miglustat use showed a protective effect. In the pediatric subgroup (*n* = 98; age < 20; 346 visits), an analysis of silent aspiration revealed that lower body weight percentiles were associated with higher odds of silent aspiration. Similar to the full cohort, disease-specific characteristics remained significant predictors (Fig. 1B), though miglustat use did not show a protective effect.

**Fig. 2:**
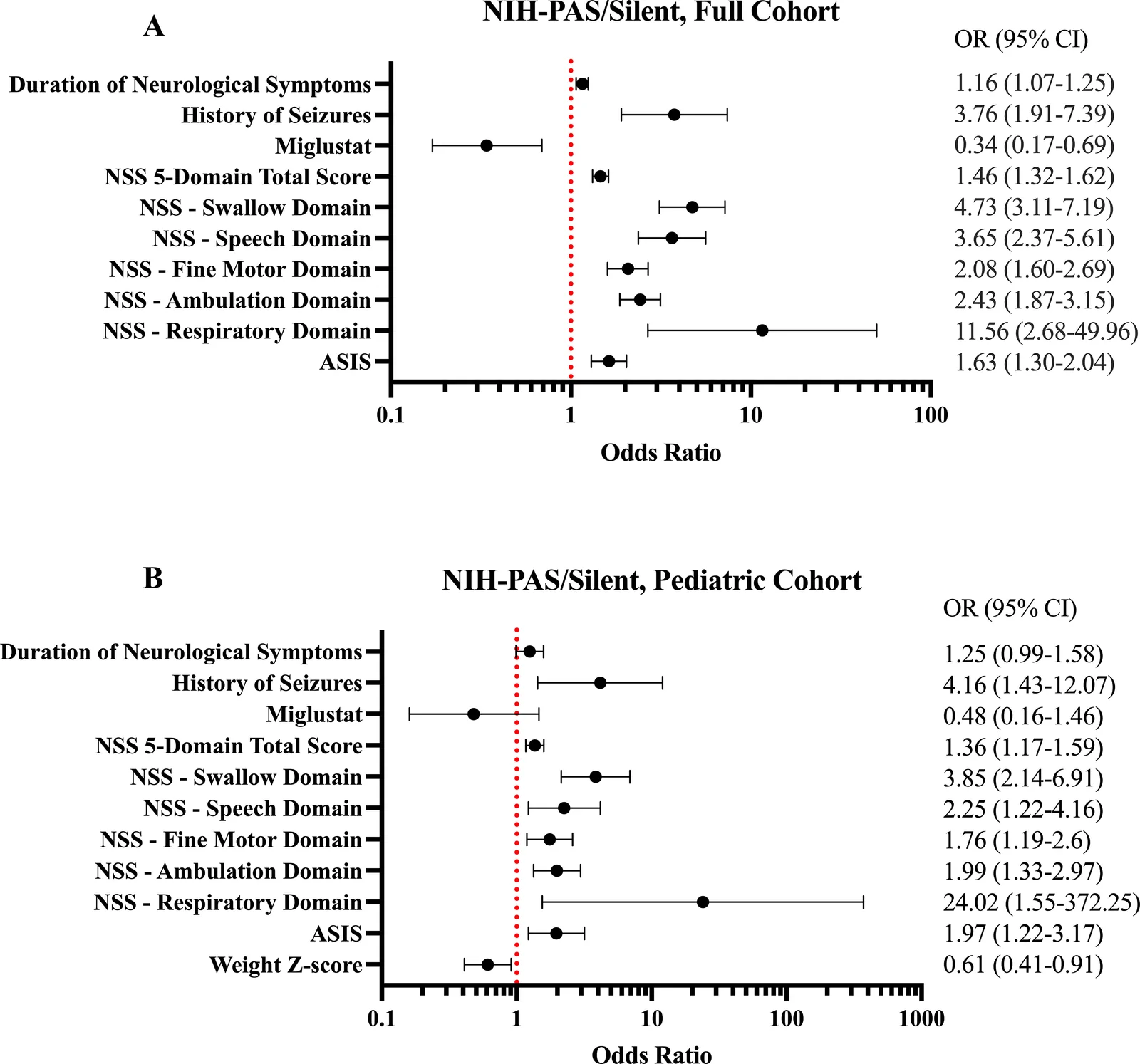
Longitudinal associations between NPC1 characteristics and NIH-PAS > 1 with or without silent aspiration. **A** represents the full cohort, and **B** represents the pediatric cohort. Forest plots display adjusted odds ratios (ORs) with 95% confidence intervals derived from longitudinal models assessing the probability of silent aspiration. Legend: Annualized Severity Increment Score (ASIS), NIH-penetration aspiration scale (NIH-PAS), Niemann-Pick Disease type C1 (NPC1), Niemann Pick Severity Scale (NSS), Odds Ratio (OR)

Additional modeling examined the role of disease-specific characteristics of developing a NIH-PAS > 1 with or without silent aspiration (Fig. 2). Results mirrored the initial analysis: longer neurological symptom duration, seizure history, and higher NSS and ASIS scores were associated with increased risk (Fig. 2A). Miglustat remained protective in the full cohort. In the pediatric group, the same risk factors were observed as well as lower body weight was associated with higher odds of a NIH-PAS > 1 and silent aspiration, but without a significant effect from miglustat (Fig. 2B).

### Time to silent aspiration survival probability

**Fig. 3:**
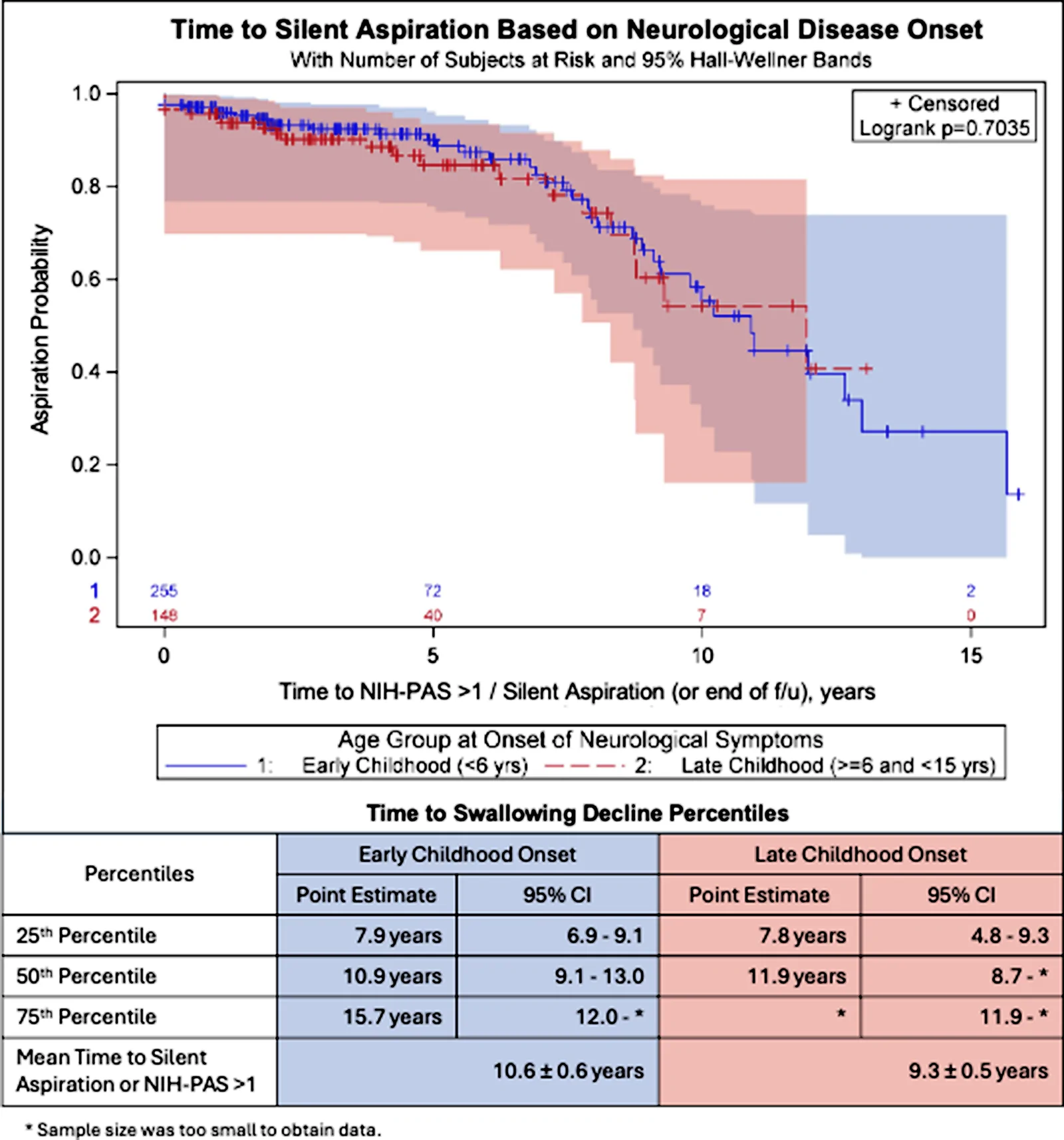
Time to silent aspiration by sub-group (early childhood onset and late childhood onset). represents Kaplan–Meier curves for time to silent aspiration (NIH-PAS > 1) stratified by early (< 6 years) vs. late (6–<15 years) childhood onset. No differences based on neurological disease onset (log-rank *p* = 0.7035). Table summarizes percentiles and mean time; estimates for later percentiles are limited by small sample size

We conducted a survival analysis of time to silent aspiration among all patients with neurological disease onset (ECO, LCO, and AO), with an overall mean time to silent aspiration was 10.3 ± 0.3 years. A secondary analysis compared ECO and LCO subgroups, excluding AO due to only one patient with documented aspiration. The mean time to silent aspiration was 10.3 ± 0.4 years for ECO and 9.3 ± 0.5 years for LCO, with no significant in survival probability between groups (*p* = 0.8629, Fig. 3).

## Discussion

In this study, we further characterize silent aspiration in individuals with NPC1, a rare pediatric neurodegenerative disorder in which this complication remains understudied. While silent aspiration has been reported in 25–50% of individuals with dysphagia or stroke using Fiberoptic Endoscopic Evaluation of Swallowing (FEES) [24, 25], we observed a notably high rate of 18.4% (24 patients) within our NPC1 cohort based on VFSS findings, highlighting the impact of this morbidity in this population.

Aspiration in NPC1 reflects disruption of the tightly integrated sensory–motor control of swallowing, including impaired bolus propulsion, delayed laryngeal vestibule closure, and weakened cough generation—deficits that typically worsen with advancing neurologic disease and contribute to pulmonary morbidity [18, 26–28]. Silent aspiration, in particular, arises from laryngopharyngeal sensory impairment, reduced central processing, and diminished motor output, resulting in impaired detection of airway invasion and loss of reflexive airway protection. Studies in stroke and Parkinson disease demonstrate that sensory deficits substantially increase aspiration in the absence of overt clinical signs, contributing to aspiration pneumonia [25, 26, 29]. The convergence of sensory–motor dysregulation explains why advancing neurologic disease elevates aspiration risk while reducing clinical warning signs.

The temporal relationship between silent aspiration and overt aspiration is not well understood, particularly in progressive neurologic disease. Some investigators have proposed that silent aspiration may emerge early as laryngopharyngeal sensory impairment precedes frank motor failure, reducing the effectiveness of protective cough and airway-clearance reflexes [30, 31]. Conversely, others have suggested that silent aspiration may represent a later stage of swallowing impairment, reflecting advanced sensory loss superimposed on established dysphagia, in which previously symptomatic aspiration becomes clinically occult [18, 32]. Longitudinal studies in neurodegenerative populations indicate that declining neurologic function and disease severity are strongly associated with increasing rates of silent aspiration, supporting a continuum in which impaired sensation and airway protection evolve over time rather than a fixed sequence [33, 34]. Within NPC1, the high prevalence of silent aspiration observed in our cohort may therefore reflect progressive disruption of both afferent sensory pathways and brainstem–cortical motor networks essential for airway defense, underscoring the importance of repeated instrumented swallowing assessments even in the absence of overt clinical symptoms.

Both pulmonary and swallowing literature support a model in which silent aspiration results from impaired airway sensation and protective reflexes, particularly the cough reflex [23, 35]. Recurrent silent aspiration promotes chronic microaspiration and aspiration pneumonia, which may be more prevalent than overt aspiration in certain populations, underscoring the importance of instrumental evaluation for early detection.

Consistent across both cross-sectional and longitudinal models, higher NSS total and domain scores, higher ASIS scores, longer duration of neurologic symptoms, and seizure history significantly increased the likelihood of silent aspiration and aspiration severity. These findings extend prior work demonstrating linear neurologic progression in NPC1 and reinforce swallowing impairment as an integral manifestation of global disease burden [19, 20].

The association between seizure history and aspiration risk may reflect both disease severity and medication-related effects on cognitive status, coordination, and airway protection, emphasizing the need for heightened vigilance in this subgroup. Patients taking reflux or gastrointestinal medications may be at an increased risk of silent aspiration. We hypothesize that this finding is not attributable to the medications themselves but rather reflects underlying gastroesophageal reflux disease, a known risk factor for aspiration and a common morbidity in neurodegenerative and neuromuscular diseases [36].

Miglustat use demonstrated a protective effect against silent aspiration in the full cohort, consistent with prior reports showing stabilization of swallowing and neurologic function in treated individuals [14]. However, this effect was not consistent in the pediatric subgroup. Our findings are more consistent with prior work by Héron et al. (2023), which suggests that miglustat does not significantly affect neurologic outcomes or survival in patients with early childhood–onset NPC1 [37]. We interpret the pediatric findings with caution, as uneven sample distribution, inconsistent follow-up, and disease progression may have confounded the observed associations. Additionally, while baseline miglustat use was documented, we did not assess ongoing treatment adherence, which may have influenced its protective effect in this population.

In our pediatric cohort, lower percentile body weight was associated with a higher prevalence of aspiration. This relationship likely reflects progressive neurologic dysfunction leading to impaired swallowing safety. These findings suggest that longitudinal weight monitoring may serve as a useful clinical marker of disease progression, facilitating earlier nutritional and swallowing evaluations with timely interventions such as dietary modification or enteral supplementation.

Our time-to-event analyses suggest that silent aspiration in NPC1 typically develops approximately a decade after neurologic symptom onset. These findings align with prior natural history studies demonstrating linear neurologic decline in NPC1 [19, 20] and extends earlier work by Solomon et al. [38], which showed that swallowing impairment and response to miglustat track closely with overall disease burden in this cohort [14]. Together, these findings underscore the importance of proactive, longitudinal instrumental swallowing surveillance.

### Limitations

This study has several limitations. NPC1 is a rare and clinically heterogeneous disorder, with substantial variability in the age of neurological onset and rate of disease progression, which may limit the generalizability of these findings. In addition, because the study cohort predominantly comprised patients with early childhood-onset and late childhood-onset neurological disease, the findings may be less representative of individuals with the adult-onset neurological form of NPC1. VFSS was performed only when clinically indicated rather than at standardized intervals, introducing the potential for selection bias and the possibility of under- or overestimating the prevalence of silent aspiration. Furthermore, instrumental swallowing assessments represent discrete time points and may not fully capture fluctuations in swallowing function over time. Although recently approved disease-modifying therapies may influence swallowing function and aspiration risk, their impact was not evaluated in this study. Despite these limitations, this study provides the largest and most comprehensive longitudinal characterization of silent aspiration in NPC1 to date and identifies clinically relevant risk factors that may facilitate earlier recognition, targeted surveillance, and timely intervention to reduce aspiration-related morbidity.

### Clinical implications

Our findings support proactive swallowing surveillance in patients with NPC1, including consideration of routine instrumental evaluations (VFSS or FEES) even in the absence of overt clinical symptoms, particularly as neurologic severity increases. Increased surveillance may be warranted among patients with increasing NSS or higher ASIS scores, seizure history, declining weight percentiles in children, gastroesophageal reflux disease, or changes in speech, ambulation, or fine motor function. Given the high prevalence of silent aspiration, reliance on bedside clinical signs alone may be insufficient to exclude aspiration risk. These results also support incorporation of swallowing outcomes as standard endpoints in NPC1 clinical monitoring and therapeutic trials. Optimal management likely requires an interdisciplinary approach with attention to modifiable contributors such as reflux control, nutritional optimization, and medication-related effects. Early dysphagia interventions, including dietary adjustments, compensatory strategies, and caregiver education, along with continuation of disease-modifying therapies, may help preserve swallowing function during neurologic decline. Education of families and caregivers regarding the potential for silent aspiration and early signs of respiratory compromise is essential to prompt timely evaluation.

## Conclusions

Silent aspiration is common in NPC1, affecting nearly one in five patients and over 20% when overt events are included. This occurs across neurological onset groups, highlighting that aspiration risk is driven by disease severity rather than phenotype. Patients with elevated NSS domain and ASIS scores, longer symptom duration, seizure history, and lower pediatric body weight are particularly vulnerable, and reflux or gastrointestinal disease further increases the risk of aspiration. Generally, miglustat use was associated with reduced risk, and silent aspiration typically emerges around 10 years after neurologic onset, providing a window for anticipatory monitoring.

Clinically, these findings support routine, proactive instrumental swallowing assessments, early dysphagia interventions, nutritional and gastrostomy management, and interdisciplinary coordination to reduce airway compromise and improve patient outcomes. These results provide clinically meaningful endpoints for future NPC1 therapeutic trials. As disease-modifying therapies emerge, further research should examine whether early detection of silent aspiration, targeted dysphagia interventions, and pharmacologic treatments can improve pulmonary outcomes and survival. Incorporating aspiration metrics as standardized endpoints will be critical for evaluating treatment effects on morbidity and quality of life in rare diseases.

Importantly, this study represents a successful, longitudinal examination of overt and silent aspiration in NPC1 using VFSS, yielding new insights into swallowing safety in this population. These results underscore the value of systematic swallowing assessments, including overt and silent aspiration, as meaningful markers of disease progression in rare disorders.

**Table 1:** Baseline characteristics of NPC1 individuals with and without silent aspiration

NPC1 Baseline Characteristics	No Silent Aspiration at any point in time	Silent Aspiration at any point in time	*p*-value
	*n*=106	*n*=24	
**Sex**, *n (%)*			
Female	55 (51.9)	14 (58.3)	0.65
Male	51 (48.1)	10 (41.7)	
**Age of Neurological Onset**, *years*, Median (IQR)*	5.0 (2.0 – 11.0)	5.5 (1.6 – 8.5)	0.49
**Weight**, *kg*,* Median (IQR)*	30.8 (14.6 – 62.1)	49.2 (25.7 – 62.0)	0.21
**Body Mass Index (BMI)**, *Median (IQR)*	18.9 (16.7 – 23.9)	21.8 (15.9 – 23.2)	0.96
**Reflux/GI Medication Use**,** *n (%)*	13 (12.4)	7 (30.4)	0.05
**Seizure Medication Use**, *n (%)*	15 (14.3)	5 (21.7)	0.36
**Miglustat Use**, *n (%)*	40 (37.7)	8 (33.3)	0.82
**NIH-PAS**, *n (%)*			**<0.0001**
0: normal	92 (86.8)	11 (45.8)	
1: Minimal Risk w/ intermittent laryngeal penetration and retrograde excursion on 1< textures	13 (12.3)	2 (8.3)	
2: Moderate- contrast enters the airway consistently on 1< textures	0 (0)	1 (4.17)	
3: Severe Aspiration	1 (0.9)	4 (16.7)	
4: Aspiration	0 (0)	6 (25)	
**NSS 5 – Domain Total Score**, *Median (IQR)*	5.0 (1.0 – 9.0)	10 (7.0 – 19.0)	**<0.0001**
**NSS – Swallow Domain**, *n (%)*			**<0.0001**
0: Normal, no dysphagia	60 (56.6)	3 (12.5)	
1: Cough while eating	17 (16.0)	6 (25.0)	
Intermittent dysphagia***	14 (13.2)	6 (25.0)	
+1 w/ liquids			
+1 w/ sollids			
Dysphagia**	9 (8.5)	2 (8.3)	
+1 w/ liquids			
+1 w/ sollids			
4: Nasogastric or gastric tube for supplemental feeding	6 (5.7)	1 (4.2)	
5: Nasogastirc or gastric tube feeding only	0 (0)	6 (25.0)	
**NSS – Speech Domain**,**** *n (%)*			**<0.0001**
0: Normal speech	46 (43.4)	1 (4.4)	
1: Mild dysarthria	50 (47.2)	11 (47.8)	
2: Severe dysarthria	7 (6.6)	5 (21.7)	
3: Non-verbal/function communications skills for needs	3 (2.8)	5 (21.7)	
5: Absence of communication	0 (0)	1 (4.4)	
**NSS – Fine Motor Domain**,***** *n (%)*			**<0.0001**
0: normal	40 (38.5)	1 (4.4)	
1: slight dysmetria/dystonia	29 (27.9)	5 (21.7)	
2: mild dysmetria/dystonia	20 (19.2)	8 (34.8)	
4: moderate dysmetria/dystonia	15 (14.2	5 (21.7)	
5: severe dysmetria/dystonia	0 (0)	4 (17.4)	
**NSS – Ambulation Domain**, ****** *n (%)*			**<0.0001**
0: Normal	33 (31.7)	2 (8.7)	
1: Clumsy	43 (41.4)	5 (21.7)	
2: Ataxic unassisted gait or not walking by18 months	14 (13.5)	6 (26.1)	
4: Assisted ambulation or not walking by 24 months	11 (10.6)	6 (26.1)	
5: Wheelchair dependent	3 (2.9)	4 (17.4)	
**NSS – Respiratory Domain**,******* *n (%)*			0.80
0: No problems	81 (89.0)	18 (85.7)	
1: History of pneumonia	9 (9.9)	3 (14.3)	
2: Pneumonia ≥ 2x/year or active therapeutic intervention	1 (1.1)	0 (0)	
**ASIS**, *Median (IQR)*	0.8 (0.3 – 1.3)	1.3 (0.9 – 2.4)	**0.0006**

*Among the 24 patients with silent aspiration, 13 (54.2%) were ECO, 10 (41.7%) were LCO, and 1(4.2%) was AO. In comparison, among the 106 patients without silent aspiration, 6 (4.6%) not yet manifested neurological symptoms, 53 (40.8%) were ECO, 32 (24.6%) were LCO, and 15 (11.5%) were AO intermittent dysphagia**

***Score is additive within these two subsections

****NSS speeech domain was available for 106 patients without silent aspiration and 23 with silent aspiration

*****NSS fine motor domain was available for 104 patients without silent aspiration and 23 with silent aspiration

*****NSS fine motor domain was available for 104 patients without silent aspiration and 23 with silent aspiration

******NSS ambulation domain was available for 104 patients without silent aspiration and 23 with silent aspiration

*******NSS respiratory domain was available for 91 patients without silent aspiration and 21 with silent aspiration

Legend

Term: Definition, IQR: inter-quartile range, the range between the 25th and 75th percentiles, ASIS: annual severity increment score, calculated by dividing the 17-domain NPC-NSS for a given visit by the age of the participant at that visit, NSS: neurological severity scale, referring to the NPC-NSS which is a 17-domain scale utilized to quantify disease severity in patients with NPC1, NSS 5-Domain Score: the 5-domain NSS consists of the ambulation, speech, swallow, fine motor, and cognition subdomains of the larger 17-domain NSS. These five domains have been shown to be a strong measure of disease severity in NPC1, NIH-PAS: NIH penetration aspiration scale, a 5 point Likert scale utilized by speech and language pathologists to quantify risk of aspiration subsequent to videofluoroscopic barium swallow studies, ASHA-NOMS: refers to the American-Speech-Language-Hearing-Association National Outcomes Measurement System, a 7 point scale utilized to measure swallowing function subsequent to videofluoroscopic barium swallow studies

**Table 2:** Baseline characteristics of NPC1 individuals with NIH PAS scores and aspiration

NPC1 Baseline Characteristics	NIH-PAS ≤ 1 & no silent aspiration during study	NIH-PAS > 1 & silent aspiration during study	*p*-value
	*n*=103	*n*=27	
**Sex**, *n (%)*			0.83
Female	54 (52.4)	15 (55.6)	
Male	49 (47.6)	12 (44.4)	
**Age of Neurological Onset**, *years*, Median (IQR)*	5.0 (2.0 – 11.0)	5.0 (1.5 – 9.0)	0.32
**Weight**, *kg*,* Median (IQR)*	31.6 (15.2 – 62.9)	45.7 (24.7 – 59.1)	0.35
**Body Mass Index (BMI)**, *Median (IQR)*	18.8 (16.6 – 24.0)	21.7 (16.1 – 23.1)	0.97
**Reflux/GI Medication Use**,** *n (%)*	13 (12.8)	7 (26.9)	0.13
**Seizure Medication Use**,*** *n (%)*	14 (13.7)	6 (23.1)	0.24
**Miglustat Use**, *n (%)*	38 (36.9)	10 (37.0)	1
**NIH-PAS**, *n (%)*			**<0.0001**
0: normal	90 (87.4)	13 (48.2)	
1: Minimal Risk w/ intermittent laryngeal penetration and retrograde excursion on 1< textures	13 (12.6)	2 (7.4)	
2: Moderate- contrast enters the airway consistently on 1< textures	0 (0)	1 (3.7)	
3: Severe Aspiration	0 (0)	5 (18.5)	
4: Aspiration	0 (0)	6 (22.2)	
**NSS 5 – Domain Total Score**, *Median (IQR)*	5.0 (1.0 – 9.0)	10 (7.0 – 19.0)	**<0.0001**
**NSS – Swallow Domain**, *n (%)*			**<0.0001**
0: Normal, no dysphagia	58 (56.3)	5 (18.5)	
1: Cough while eating	17 (16.5)	6 (22.2)	
Intermittent dysphagia****	14 (13.6)	6 (22.2)	
+1 w/ liquids			
+1 w/ sollids			
Dysphagia***	9 (8.7)	2 (7.4)	
+1 w/ liquids			
+1 w/ sollids			
4: Nasogastric or gastric tube for supplemental feeding	5 (4.9)	2 (7.4)	
5: Nasogastirc or gastric tube feeding only	0 (0)	6 (22.2)	
**NSS – Speech Domain**,***** *n (%)*			**<0.0001**
0: Normal speech	46 (44.7)	1 (3.9)	
1: Mild dysarthria	48 (46.6)	13 (50.0)	
2: Severe dysarthria	7 (6.8)	5 (19.2)	
3: Non-verbal/function communications skills for needs	2 (1.9)	6 (23.1)	
5: Absence of communication	0 (0)	1 (3.9)	
**NSS – Fine Motor Domain**,****** *n (%)*			**0.0001**
0: normal	38 (37.6)	3 (11.5)	
1: slight dysmetria/dystonia	29 (28.7)	5 (19.2)	
2: mild dysmetria/dystonia	20 (19.8)	8 (30.8)	
4: moderate dysmetria/dystonia	14 (13.9)	6 (23.1)	
5: severe dysmetria/dystonia	0 (0)	4 (15.4)	
**NSS – Ambulation Domain**,******* *n (%)*			**<0.0001**
0: Normal	32 (31.7)	3 (11.5)	
1: Clumsy	43 (42.6)	5 (19.2)	
2: Ataxic unassisted gait or not walking by18 months	13 (12.9)	7 (26.9)	
4: Assisted ambulation or not walking by 24 months	11 (10.9)	6 (23.1)	
5: Wheelchair dependent	2 (2.0)	5 (19.2)	
**NSS – Respiratory Domain**,******** *n (%)*			1
0: No problems	78 (88.6)	21 (87.5)	
1: History of pneumonia	9 (10.2)	3 (12.5)	
2: Pneumonia ≥ 2x/year or active therapeutic intervention	1 (1.1)	0 (0)	
**ASIS**, *Median (IQR)*	0.8 (0.3 – 1.3)	1.3 (0.8 - 2.5)	**0.0006**

*27 patients with NIH-PAS >1 and silent aspiration, 15 (55.6%) had ECO, 11 (40.7%) had LCO, and 1 (3.7%) had AO. In comparison, among the 103 patients with NIH-PAS scores ≤ 1, 6 (5.8%) were neurologically asymptomatic, 51 (49.5%) had ECO, 31 (30.1%) had LCO, and 15 (14.6%) had AO

**Usage of reflux/GI medications was only available for 102 patients with PAS ≤ 1 and without silent aspiration and 26 with PAS >1 or silent aspiration

***Usage of seizure medications was only available for 102 patients with PAS ≤ 1 and without silent aspiration and 26 with PAS >1 or silent aspiration

****Score is additive within these two subsections

*****NSS speech domain was available for 103 patients with PAS ≤ 1 and without silent aspiration and 26 with PAS >1 or silent aspiration

******NSS fine motor domain was available for 101 patients with PAS ≤ 1 and without silent aspiration and 26 with PAS >1 or silent aspiration

*******NSS ambulation domain was available for 101 patients with PAS ≤ 1 and without silent aspiration and 26 with PAS >1 or silent aspiration

********NSS respiratory domain was not available for all patients: 88 with PAS ≤ 1 and without silent aspiration and 24 with PAS >1 or silent aspiration

Legend

Term: Definition, IQR: inter-quartile range, the range between the 25th and 75th percentiles, ASIS: annual severity increment score, calculated by dividing the 17-domain NPC-NSS for a given visit by the age of the participant at that visit, NSS: neurological severity scale, referring to the NPC-NSS which is a 17-domain scale utilized to quantify disease severity in patients with NPC1, NSS 5-Domain Score: the 5-domain NSS consists of the ambulation, speech, swallow, fine motor, and cognition subdomains of the larger 17-domain NSS. These five domains have been shown to be a strong measure of disease severity in NPC1, NIH-PAS: NIH penetration aspiration scale, a 5 point Likert scale utilized by speech and language pathologists to quantify risk of aspiration subsequent to videofluoroscopic barium swallow studies. ASHA-NOMS: refers to the American-Speech-Language-Hearing-Association National Outcomes Measurement System, a 7 point scale utilized to measure swallowing function subsequent to videofluoroscopic barium swallow studies

## Data Availability

The data that support the findings of this study are not openly available due to reasons of sensitivity and are available from the corresponding author upon reasonable request. Data are located in controlled access data storage at NIH.

## Declarations

This research was supported [in part] by the Intramural Research Program of the National Institutes of Health (NIH). The contributions of the NIH author(s) are considered Works of the United States Government. The findings and conclusions presented in this paper are those of the author(s) and do not necessarily reflect the views of the NIH or the U.S. Department of Health and Human Services.

## Abbreviations

AO: Adult onset
ASIS: Annualized Severity Increment Score
BMI: Body mass index
FDA: Food and Drug Administration
FEES: Fiberoptic Endoscopic Evaluation of Swallowing
ECO: Early childhood onset
LCO: Late childhood onset
NIH: National Institutes of Health
NIH-PAS: NIH-penetration aspiration scale
NPC1: Niemann-Pick Disease type C1
NSS: NPC Neurological Severity Score
SLP: Speech-language pathologist
VFSS: Videofluoroscopic swallow studies
